# Supplementary material for: Factors associated with COVID-19 vaccination schedule completion among adults in high-social-vulnerability neighborhoods in two Brazilian state capitals: A cross-sectional study
Source: PLoS One. 2026 Apr 3;21(4):e0346091. doi: 10.1371/journal.pone.0346091 (PMC13048420; doi:10.1371/journal.pone.0346091)
Supplement: S1 Table — (DOCX) [file pone.0346091.s001.docx]

### Supplementary material

**S1 Table. Multilevel logistic regression analysis of factors associated with COVID-19 vaccination schedule completion among adults in two Brazilian state capitals, 2022–2023 (TQT-COVID-19 Study; N = 6,827).**

| **Variables (Fixed Effects)** | **OR (95%CI)** |
| --- | --- |
| **Age** |  |
| 18–30 years | 1 |
| 31–50 years | 1.51 (1.31–1.73) |
| >50 years | 3.80 (3.09–4.68) |
| **Gender identity** |  |
| Cisgender man | 1 |
| Cisgender woman | 1.38 (1.21–1.58) |
| **Education level** |  |
| Up to elementary/middle school | 1 |
| Complete or incomplete high school | 1.35 (1.15–1.58) |
| Higher education and graduate | 2.38 (1.94–2.93) |
| **Evangelical religion** |  |
| No | 1 |
| Yes | 0.64 (0.57–0.73) |
| **Density of people per room** |  |
| ≤0.5 | 1 |
| 0.5–0.99 | 1.02 (0.88–1,18) |
| >=1 | 0.70 (0.58–0.84) |
| **Number of comorbidities** |  |
| None | 1 |
| Only one | 1.34 (1.13–1.58) |
| Two or more | 2.13 (1.56–2.91) |
| **Forms of access to health services** |  |
| Exclusively through SUS (Public) | 1 |
| Private | 1.32 (1.06–1.65) |
| Public and private | 1.53 (1.27–1.85) |
| **Last medical consultation** |  |
| More than 12 months or never | 1 |
| In the last 12 months | 1.61 (1.39–1.86) |
| **Sought care at the health unit in the last 12 months** |  |
| No/appointment could not be scheduled | 1 |
| Yes, and received care | 1.20 (1.02–1.40) |

**Random effects (UBS level):** variance of intercept = 0.038; standard deviation = 0.195; intraclass correlation coefficient (ICC) = 0.012.

OR = odds ratio; CI = confidence interval; ICC = intraclass correlation coefficient
