# Supplementary material for: Factors associated with COVID-19 vaccination schedule completion among adults in high-social-vulnerability neighborhoods in two Brazilian state capitals: A cross-sectional study
Source: PLoS One. 2026 Apr 3;21(4):e0346091. doi: 10.1371/journal.pone.0346091 (PMC13048420; doi:10.1371/journal.pone.0346091)
Supplement: Attachment — Submitted filename: Answer__to_reviewers_PONE-D-25-35448.docx [file pone.0346091.s003.docx]

December 12, 2025

Editor Vinícius Silva Belo

Editorial Board

Plos One

Ref.: Manuscript ID PONE-D-25-35448 entitled " Factors associated with COVID-19 vaccination schedule completion among adult in high social vulnerability neighborhoods in two Brazilian state capitals: A cross-sectional study"

Dear Editorial Board Members:

We would like to thank you for the opportunity to resubmit a revised copy of our manuscript “Factors associated with COVID-19 vaccination schedule completion among adults in high-social-vulnerability neighborhoods in two Brazilian state capitals: A cross-sectional study”. We would also like to express our gratitude to the reviewers for their feedback and helpful comments regarding corrections or modifications.

Please find below our answers to the reviewers' comments:

Reviewer #1: The manuscript addresses an important topic and provides valuable insights regarding vaccination uptake in socially vulnerable populations. However, several methodological and presentation issues require careful revision to strengthen the validity and clarity of the findings.

Minor Methodological Limitations

Definition of the outcome

The study defined a complete vaccination schedule as the primary series plus two booster doses. However, during the data collection period, not all participants were eligible for two boosters (depending on age, risk group, and national guidelines). This may have led to misclassification of individuals as “incomplete,” even if they were up to date according to contemporaneous recommendations. Requiring two boosters for all participants risks classifying as “incomplete” those who were not eligible at the time (due to age, risk profile, interval since last dose, or guideline changes). As a reflection, the outcome should be redefined as “adherence according to eligibility” (age/conditions + guidelines in force at the date of interview) and analyses repeated.

**Answer:** Thank you for these comments. We included a figure with the COVID-19 vaccination timeline in Brazil, describing the start of the campaign and the progressive inclusion of different groups. For this reason, we analyzed the data considering a complete vaccination schedule as the primary series plus at least one booster dose, rather than two boosters, because during the data collection period, all participants aged 18 years or older were eligible for the primary series and at least one booster dose.

Inadequate handling of missing data

Table 4 reports N=6,805, lower than the total of vaccinated participants with at least one dose (7,193), suggesting a complete-case analysis without description of missing-data handling. The absence of a clear strategy for managing missing values may introduce systematic bias. It is recommended to report the pattern and proportion of missing data by variable and conduct sensitivity analyses.

**Answer:** Thank you for this suggestion. In Table 1 we included the number of participants with complete information for each variable. We excluded participants with missing information, as stated in the Methods section.

Inconsistencies and Critical Data Presentation Issues

Reversed percentages in Table 3 header: listed as “Yes (n=3,080, 57.2%) / No (n=4,113, 42.8%).” However, 3,080/7,193=42.82%, which is consistent with the distribution in Table 1 (“up to second booster” = 42.82%). The header percentages have been corrected accordingly.

**Answer:** We revised it accordingly.

Inconsistent numeric notation: mixed use of separators (e.g., “28,52” and “2.882” in Table 3), which may confuse readers and hinder verification. All tables should be standardized—using a dot for decimals and consistent thousand separators. The problem recurs across tables. Recommendation: apply a consistent numeric format (e.g., decimal point; thousand separator as comma or space).

**Answer:** We revised it accordingly.

Key corrections and additions include:

1. Correct the Table 3 header (reversed percentages) and standardize numeric notation across all tables.

**Answer:** We have made the corrections.

2. Redefine the outcome based on eligibility criteria and reanalyze, retaining the current definition as a sensitivity analysis.

**Answer:** We redefined our outcome variables based on the availability criteria and reanalyzed the data.

3. Address missing data (describe patterns; consider multiple imputation; include income with “not reported” as a category). The analyzed sample size (N) for each model is reported.

**Answer:** We included the total sample size (N) for each variable and model in the tables and included the missing data in the income variable as a limitation; however, we had other variables to assess socioeconomic factors.

4. Include a timeline of booster recommendations (by age and risk group), indicating the proportion of participants eligible in each stratum.

**Answer:** We included a figure showing the COVID-19 vaccination timeline for Brazil. Based on this timeline, individuals aged 18 years and older were already eligible to receive a booster dose before the study’s data collection period. Therefore, we considered the vaccination schedule to be complete for individuals receiving one or two booster doses and did not find it necessary to include the proportion of participants eligible for each stratum.

Reviewer #2: General Assessment

The manuscript addresses an important subject that is the factors associated with COVID-19 vaccination schedule completion in socially vulnerable neighborhoods in two Brazilian capitals. The study benefits from a large sample size, standardized data collection, and the inclusion of two distinct settings. However, several clarifications, additions, and structural adjustments would strengthen the manuscript and make the findings more transparent.

Major Points

Study Population and Flow Diagram

Although the questionnaire was reportedly administered to individuals aged ≥12 years, the analysis only includes participants aged ≥18 years. Please clarify this point. How many were excluded? I recommend including a flow diagram showing the number of individuals invited, excluded (<18 years) and retained for analysis. In addition, consider including in the supplementary material the full questionnaire used in data collection.

**Answer:** Thank you for your comments and suggestions. We included a flow chart showing the number of individuals invited, excluded, and retained in the analysis. Individuals under 18 years of age were excluded because they were not eligible for the full vaccination schedule. The complete questionnaire used in the study has been added as an appendix to the publication of the protocol (Reference 18 in our article).

Study Setting

The manuscript lacks a dedicated paragraph in the Methods section describing the study setting. I recommend that the authors clearly describe the healthcare units selected for data collection as well as the municipalities and neighborhoods included in the analysis. Providing this information would allow readers to better understand the context, representativeness and potential differences between the study sites.

**Answer:** We included information describing the study setting and health care units selected for data collection, which are also available in the protocol published in reference 18.

Vaccine Type, Timing and Adherence

The data collection instrument reportedly captured vaccine type. In Brazil, multiple vaccine schemes were implemented depending on age, comorbidities and city. Different vaccines were subject to distinct public perceptions and adverse event profiles, for example controversy surrounding CoronaVac and Pfizer and reports of post vaccination side effects. Please consider either analyzing or at least discussing whether vaccine type may have influenced completion of the second dose or booster uptake.

**Answer:** We collected information on vaccine type; however, this variable was subject to considerable recall bias and missing data, as it was self-reported rather than obtained from vaccination cards. We did not collect information regarding the reasons for not completing the vaccination schedule; however, we agree that this information would have been valuable. We have added this information in the Discussion.

If vaccination dates for the study population were recorded, please make this explicit in the manuscript. If these dates are available, it is important to indicate whether all participants had equal opportunity to complete the vaccination schedule during the study period. For example, older adults and individuals with comorbidities may have had more time to receive boosters than younger adults. Likewise, individuals who began their vaccination scheme close to the time of questionnaire administration might not have had the opportunity to complete the schedule. Please place the timing of vaccination of the study population in the context of the national and local vaccination campaigns. This context helps explain some of the observed associations. It would also be useful to comment on possible differences in campaign implementation between Salvador and Rio de Janeiro, given the higher completion rate in Rio.

**Answer:** The variables containing the dates of the vaccines received by the participants also had many missing values, making it impossible to perform the analyses. To improve the visualization of the eligibility of the population aged 18 years and older for COVID-19 vaccination, we added a figure showing the COVID-19 vaccination timeline in Brazil, describing the start of vaccination and the inclusion of different groups. For this reason, we reanalyzed the data considering a complete vaccination schedule as the primary series plus at least one booster dose, rather than two boosters, because during the data collection period, all participants aged 18 years or older were eligible for the primary series and at least one booster dose. We have also added a brief description of the Salvador and Rio de Janeiro municipalities to the Methods section .

Use of SUS vs. Private Health Services

The title of the manuscript implies that that the study population are only adult users of primary health care. Does part of the study population exclusively use health insurance or private services? If so, the title may not accurately describe the total sample. Please clarify this point. My uncertainty comes from the following description: “Access to health services: Forms of access to health services (exclusively through SUS, health insurance and private, all services).” Please explain clearly what “all services” means. Also, the journal has an international audience, it would be helpful to describe item (iii) more explicitly as there are terms that only Brazilian readers would easily understand.

**Answer:** We agree that not all the participants were users of primary healthcare services. Therefore, we updated the title to: “Factors associated with COVID-19 vaccination schedule completion among adults in high-social-vulnerability neighborhoods in two Brazilian state capitals: A cross-sectional study.” We also redefined the variable “forms of access to health services” to clarify that “all services” refer to both public and private services. In addition, we provided a clearer explanation of item (iii) to an international audience.

Also, Table 4 shows that exclusive SUS users had lower odds of completing the vaccination schedule than those with private, if I clearly understood, or mixed public–private use (all services??). The Discussion should explicitly acknowledge this distinction and avoid overstating PHC’s role beyond what the data support, as already indicated in the first sentence of the Conclusion. The statement “The study demonstrated that PHC plays a pivotal role…” implies causality. A more accurate formulation would be: “Our findings indicate that identifying PHC as the usual source of care is associated with higher odds of completing the vaccination schedule, suggesting that PHC may facilitate adherence; however, exclusive use of SUS services showed lower completion rates.”

**Answer:** We have accepted your suggestions and revised the Discussion section accordingly. We explicitly acknowledge the distinction between exclusive SUS users and those using private or public and private services. We have rephrased the statement about PHC to reflect associations rather than causality, as recommended.

Data Visualization

The manuscript relies heavily on tables and would benefit from more intuitive graphics. I suggest including a forest plot displaying adjusted odds ratios from the multivariable model. Also, maybe a stacked bar chart showing vaccination stages by city, age group, sex and religion,

Additionally, Table 2 appears redundant because Table 3 already presents counts and percentages in the context of the analysis. If the authors wish to retain the total numbers, they could add the total N and percentage in a third column rather than keeping a separate table.

**Answer:** We have included this figure in place of Table 1. We also included sample sizes for each variable.

Discussion Structure

I recommend restructuring the Discussion to begin with a succinct paragraph summarizing the main quantitative findings (odds ratios). It is also very important to include a dedicated paragraph on the limitations of the study, such as the self-reported nature of vaccination data. If no analysis by vaccine type is presented, this should be acknowledged as a limitation as well. The cross-sectional design should also be mentioned because it precludes causal inference. In addition, the Discussion could emphasize the strengths of the study, such as the large sample of PHC users in high vulnerability areas, the use of a standardized questionnaire and the employment of trained interviewers.

**Answer:** We have revised the Discussion accordingly.
