# Supplementary material for: Factors associated with COVID-19 vaccination schedule completion among adults in high-social-vulnerability neighborhoods in two Brazilian state capitals: A cross-sectional study
Source: PLoS One. 2026 Apr 3;21(4):e0346091. doi: 10.1371/journal.pone.0346091 (PMC13048420; doi:10.1371/journal.pone.0346091)
Supplement: Attachment — Submitted filename: Response Editor Comment_LM.docx [file pone.0346091.s004.docx]

**Comment**

Although the reviewers considered the authors’ responses to most comments satisfactory, an important methodological point raised in the first round of review was not addressed. The following comment, which was also provided in the initial evaluation, should be carefully considered and explicitly addressed in the revised manuscript:

Given the hierarchical structure of the data across health units, it is necessary analyze the data using multilevel models, as standard logistic regression may underestimate standard errors and overlook variability between units. In addition, the potential for collinearity among the modeled variables should be formally assessed and, if present, corrected. A discussion on the statistical power of variables with small numbers of participants is also required, as well as a more in-depth discussion on the external validity of the findings.

**Response**

Dear Editor,

We sincerely apologize for having inadvertently overlooked this important methodological comment raised during the first round of review. We appreciate your careful attention in bringing this issue back to our notice.

Thank you for the suggestion to consider a multilevel modeling approach. We carefully evaluated a random-effects logistic regression including UBS as a random intercept and compared its performance with the standard logistic regression model.

The intraclass correlation coefficient was very low (ICC = 1.2%), indicating minimal clustering of the outcome at the UBS level. In addition, odds ratios and confidence intervals were nearly identical across both models, and the substantive interpretation of the results did not change. We consider that the added complexity of the multilevel model did not provide meaningful analytical gains; for that reason, and because the core conclusions remain unchanged, we therefore chose to retain the standard logistic regression model. We included a supplementary Table S1 as a Supplementary Material, and this information in the results: “The multilevel analysis (S1 Table) showed a very low intraclass correlation coefficient (ICC = 1.2%), indicating minimal clustering of the outcome at the UBS level. Additionally, odds ratios and corresponding confidence intervals were nearly identical between the multilevel and standard logistic regression models, with no substantive changes in the interpretation of the results. Therefore, the standard logistic regression model was retained, as the main conclusions remained unchanged“.

Multicollinearity was assessed using variance inflation factors (VIF). All variables presented VIF values below 2 (mean VIF = 1.31), indicating the absence of relevant multicollinearity. We included this information in the results: “All variables presented VIF values below 2 (mean VIF = 1.31), indicating the absence of relevant multicollinearity”.

Given the large overall sample size and the high expected cell counts across the analyzed variables, the study has adequate statistical power to detect meaningful associations, and the risk of unstable estimates due to sparse data is minimal. We included the following information in the limitations: “Furthermore, external validity may be limited, as the study was based on a non-probabilistic sample recruited through health services. Consequently, caution is warranted when generalizing the results beyond similar service-based populations”.
